# GMMA-Based Vaccines: The Known and The Unknown

**DOI:** 10.3389/fimmu.2021.715393

**Published:** 2021-08-03

**Authors:** Francesca Mancini, Francesca Micoli, Francesca Necchi, Mariagrazia Pizza, Francesco Berlanda Scorza, Omar Rossi

**Affiliations:** GlaxoSmithKline (GSK) Vaccines Institute for Global Health (GVGH), Siena, Italy

**Keywords:** Generalized modules for membrane antigens, GMMA, OMV, vaccine, mode of action, reactogenicity

## Abstract

Generalized Modules for Membrane Antigens (GMMA) are outer membrane vesicles derived from Gram-negative bacteria engineered to provide an over-vesiculating phenotype, which represent an attractive platform for the design of affordable vaccines. GMMA can be further genetically manipulated to modulate the risk of systemic reactogenicity and to act as delivery system for heterologous polysaccharide or protein antigens. GMMA are able to induce strong immunogenicity and protection in animal challenge models, and to be well-tolerated and immunogenic in clinical studies. The high immunogenicity could be ascribed to their particulate size, to their ability to present to the immune system multiple antigens in a natural conformation which mimics the bacterial environment, as well as to their intrinsic self-adjuvanticity. However, GMMA mechanism of action and the role in adjuvanticity are still unclear and need further investigation. In this review, we discuss progresses in the development of the GMMA vaccine platform, highlighting successful applications and identifying knowledge gaps and potential challenges.

## Introduction

Both pathogenic and nonpathogenic Gram-negative bacteria are able to spontaneously release 25–250 nm vesicles during growth, especially during the end of log phase (1). Since they originate from the bacterial outer membrane, these vesicles reflect the membrane composition and are then named outer membrane vesicles (OMVs). They contain bacterial antigens such as lipopolysaccharides (LPS) and proteins in their original environment, and additional immunostimulatory molecules (i.e. lipoproteins, peptidoglycans). Because of their composition, they raise high scientist interest and have been widely investigated as a promising vaccine platform (2, 3).

However, bacteria naturally release OMV but in relatively low amounts, and contain endotoxins in the natural form, which might cause systemic reactogenicity in humans depending on the vaccine dose used. Hence, in order to overcome issues of limited yield and to reduce the levels of endotoxicity, bacterial strains have been genetically modified to increase outer membrane vesiculation (4–6) and reduce LPS endotoxicity (7–9). We named the resulting vesicles, deriving from the over-vesiculating strain and with mutations in the LPS genes, Generalized Modules for Membrane Antigens (GMMA) whereas others called them mutant-derived or genetically detoxified OMV. Through a three-step process consisting on fermentation of the GMMA-producing strain coupled with two consecutive tangential flow filtration steps (4), GMMA can be produced at high yield and purity through a simple process. Indeed, the entire production process from fermentation to final purified GMMA lasts 3 days and thus, depending on the size of the vaccine dose, a relatively small manufacture facility with a 500 L fermenter could produce 100 000 000 doses of vaccines per year at a manufacturing cost of approximately $1 per dose (10). GMMA from *Shigella* (4, 5, 7, 9, 11–13), *Salmonella* (8, 14, 15), and *Neisseria* (16, 18) species have been already generated using this approach, which is shown to be flexible enough to be potentially extended with minimal adjustments to any Gram-negative bacterial species. Indeed multiple industrial (17–19) and research (20–22) approaches based on genetic engineering of bacteria for hyper-vesiculating and surface-expression of a variety of homologous and heterologous antigens, including bacterial (20–22), viral (23), parasitic (24) and even cancer antigens (25) have been described.

In this review, we will refer to genetically modified OMV as GMMA, and will focus our attention on GMMA-based vaccines that are in an advanced stage of the development and already moved or are approaching to move in clinical trials rather than on research vaccines. We will discuss progress in the development of the GMMA vaccine platform, highlighting successful applications, gaps and potential challenges.

## GMMA As A Vaccine Platform

GMMA constitute a straightforward technology based on low-cost of production and high purification yields and is therefore suitable for the development of vaccines against bacterial pathogens and particularly of affordable vaccines targeting low- and middle-income countries (LMICs).

GMMA resemble faithfully the outer membrane of the bacterial pathogen they shed from but lack the ability to cause the associated disease. They present to the immune system key antigens in their natural environment and conformation, facilitating uptake by immune cells and inducing strong immune response (26). The GMMA outer membrane also displays several Pathogen Associated Molecular Patterns (PAMPs), small molecular motifs well conserved in bacteria which are recognized by Patter Recognition Receptors (PRRs) expressed on mammalian cells (27). PAMPs interaction with PRRs rapidly activates the complex signaling pathway, with the induction of pro-inflammatory cytokines and chemokines, and that may be the basis of GMMA self-adjuvanticity. However, while activation of the innate immune system can result in a high immune response to an antigen, it may also induce local and severe adverse effects in humans, from febrile response to septic shock (28). Thus, fine tuning the balance between immune stimulation and reactogenicity is key for an acceptable GMMA-based vaccine.

LPS, the most abundant component of GMMA, is a key antigen in Gram-negative bacteria, but it is also the main component for systemic reactogenicity (29). Intrinsic LPS endotoxicity can be reduced by genetically modifying the lipid A structure. Lipid A is the endotoxic component of LPS which mediates the binding to toll like receptor (TLR) 4 inducing innate immune activation. TLR4 recognition of LPS is strongly influenced by the structure of its lipid A component which, in most Gram-negative bacteria (i.e., *E. coli*, *Shigella*, *Salmonella*), is composed of a β-1’,6-linked disaccharide of glucosamine phosphorylated at the 1 and 4’ positions and acylated at 2, 3, 2’ and 3’ position with R-3-hydroxymyristate (called lipid IV A). This scaffold is then decorated in various positions with fatty acids differing in length and saturation level or with substituents of phosphoryl groups of glucosamines (30). The most reactogenic form of lipid A is the bis-phosphorylated and hexa-acylated with 12 to 14 carbon acyl chains and an asymmetric (4/2) distribution, whereas the penta-acylated lipid A is much less active than the hexa-acylated form in activating human TLR4 (31–33). Bacteria use lipid A modifications as immune evasion mechanism and to adapt to environmental changes (e.g., temperature, nutrient, osmolarity) (30, 34). The selection of the appropriate modified lipid A structure, depending on the structure present in the parent bacteria, is key to balance immunogenicity and reactogenicity of GMMA-based vaccines. Indeed, the selection of the modifications on the lipid A acylation status are critical, as most of the genes implicated in LPS biosynthesis are necessary to preserve bacterial membrane stability and therefore viability. Genes that encode for acyltransferases such as HtrB or MsbB in *Shigella* (4, 5, 7, 9), MsbB and PagP in *Salmonella* (8), or LpxL1 in *N. meningitidis* (16, 17, 35) have been mutated to generate GMMA with different penta-acylated lipid A forms.

In addition to LPS, other molecules contained in GMMA, like lipoproteins, are able to stimulate the innate immune system through the activation of TLR2. Indeed, GMMA from *Shigella* and *Salmonella* were able to stimulate peripheral blood mononuclear cells (PBMCs) and induce interleukin (IL)-6 release, which was almost completely abolished when a combination of antibodies blocking TLR4 and TLR2 was used (7, 8), indicating that they are the PRRs mostly involved in GMMA mediated cytokine release.

GMMA act as effective adjuvant and carrier, thus having an intrinsic ability to improve immunogenicity of protein and carbohydrate antigens. The glycoconjugate approach is the gold standard for enhancing immunogenicity, particularly for polysaccharides (36). Bacterial polysaccharides are T-cell-independent antigens which are generally not able to elicit germinal center (GC) formation and therefore immunological memory, persistence of antibody response, and affinity maturation of B cell receptors. Covalent linkage to a suitable carrier protein confers to saccharide antigens the ability to elicit a T-cell-dependent response, overcoming the limitation listed above. Consequently, vaccination with conjugates enhances polysaccharide immunogenicity and protective efficacy, especially in infants and children under 2 years of age (37, 38). As carriers for polysaccharides, GMMA can be somehow considered multi-valent antigens, as they may present multiple polysaccharide molecules and proteins in natural conformation (39). GMMA was shown to be superior to traditional glycoconjugate vaccines in animal models, likely due to the nano-sized particle properties, ability to present multiple saccharide epitopes and self-adjuvanticity. GMMA from *S.* Typhimurium and *S.* Enteritidis strains elicited levels of anti-O polysaccharide-specific immunoglobulins (Ig)G comparable to those observed after vaccination with corresponding CRM_197_ glycoconjugates formulated with Alhydrogel, but showed an increased IgG antibody isotype profile and improved complement-mediated bactericidal activity and protective ability in challenge model (40). Similarly, GMMA from *S. flexneri* 6 strain induced higher anti-O-antigen IgG titers compared to CRM_197_ glycoconjugate when both were administered to mice without Alhydrogel (13).

GMMA can also be carriers for heterologous antigens, which renders them even a more powerful vaccine platform. Bacterial strains can be engineered for overexpression in GMMA of key homologous and heterologous antigens or multiple antigenic variants (6, 41–43). In alternative to genetic manipulation, decoration of GMMA with heterologous polysaccharide or protein antigens can also be achieved through chemical conjugation (44).

## Successful Applications Of GMMA Vaccine Platform

Safe, cost-effective and affordable technologies such as GMMA constitute ideal vaccine candidates to prevent diseases prevalent in LMICs (10). Indeed, many GMMA-based vaccines targeting several bacterial pathogens are in development (45, 46). Currently, the most advanced GMMA-based vaccine is the *Shigella sonnei* 1790GAHB that has been extensively tested in preclinical (5) and clinical studies (47–49). *Shigella sonnei* GMMA formulated with Alhydrogel were highly immunogenic in mice and rabbits and had low stimulatory activity in the *in vitro* monocyte activation test. The low reactogenicity was also confirmed by two *in vivo* models: a modified rabbit pyrogenicity test based on the intramuscular administration of the full human dose and a multiple dose toxicology study in rabbits using intramuscular, intranasal, and intradermal administration routes (5). Moreover, *Shigella sonnei* GMMA elicited in the animal model antibodies not only against the key antigen LPS, but also against GMMA proteins (9). In phase I and II clinical studies, the vaccine was well tolerated up to 100 μg following intramuscular (two or three doses), intradermal or intranasal administrations (48). Moreover, the vaccine induced anti-LPS specific antibodies in healthy adults in Europe and in Kenya, where shigellosis is endemic (47, 48). In addition, immune response was significantly increased following a booster dose, administered two-three years after the primary immunization (49). Antibodies elicited were able to induce complement mediated bactericidal killing in a dose dependent manner (50). The promising results obtained with *Shigella sonnei* GMMA vaccine corroborated the ability of GMMA to be an ideal delivery system for *Shigella* LPS, and to induce high levels of anti-*Shigella* LPS IgG considered as the most promising correlate of protection against shigellosis (51). However, based on recent results not yet published from a controlled human infection model (CHIM) study, it seems that *Shigella* LPS amount is critical to induce a level of IgG antibodies able to protect from *Shigella* infection and additional work has been done for improving the design of a 4-component *Shigella* GMMA-based vaccine that is entering clinical testing.

An additional GMMA-based vaccine in an advanced stage of development is the HOPS-G meningococcal vaccine adjuvanted with aluminum hydroxide. GMMA were derived from an engineered meningococcal B strain, containing the deletion of the *lpxL*1 and *synX* genes to remove the expression of capsular polysaccharide and reduce LPS reactogenicity, over-expressing factor H binding protein, two PorAs and the stabilized OpcA (17). When tested in a human clinical trial as a three doses vaccine, it resulted to be safe and immunogenic. Sera form 79% of volunteers, collected two weeks after third dose had a fourold or greater increase in bactericidal activity against the homologous strain by which the GMMA were derived. 68% of volunteers showed cross-protection against strains carrying the L3-5,7-5 phase variant of the parent strain. Similar results were obtained with a vaccine differing only for the strategy used to detoxify the lipo-oligosaccharide: lipid A acylation status was indeed modified through disabling the *lpxL2* gene instead of the *lpxL*1 (18).

The results obtained so far with the GMMA-based vaccines tested in clinical trials paved the way for other GMMA-based vaccines. Indeed multi component GMMA-based vaccines consisting in GMMA derived from the key disease-causing *invasive* nontyphoidal *Salmonellae* (*Salmonella* Typhimurium and *Salmonella* Enteritidis) strains, either alone or in combination with the recently licensed Vi-CRM conjugate vaccines are moving to Phase I/II clinical studies. Results in mice showed that *S*. Typhimurium and *S*. Enteritidis GMMA were able to elicit strong and functional bactericidal anti-LPS O-antigen antibodies and a broad IgG range of antibodies. Moreover, induced antibodies were able to protect in an *in vivo* mouse challenge model (40).

## Gaps On Understanding The Basis Of GMMA Immunogenicity And Potential Challenges

Recent clinical trials not only showed good immunogenicity data but highlighted a satisfactory tolerability profile of GMMA. However, due to differences in maturation of TLR in children and adults (52), the risk of systemic reactogenicity in different age groups needs to be further evaluated, and age-de-escalation clinical trials will be necessary, especially for GMMA vaccines targeting diseases mainly affecting children.

Moreover, Alhydrogel has been used in preclinical and clinical studies in *S. sonnei* GMMA vaccine formulation as absorbent agent with the solely purpose of reducing GMMA reactogenicity, mainly due to results observed after intramuscular immunization of rabbits (29). Indeed, intramuscular immunization of rabbits with 100 µg of 1790GAHB caused only a low and transient temperature rise, comparable to what observed with 5 µg of *S. sonnei* GMMA delivered alone. However, no results in humans comparing non-adjuvanted and Alhydrogel-adsorbed GMMA are yet available. Similar preclinical results have been obtained when OMV from *Neisseria meningitidis* (*Nm*) were tested in a rabbit pyrogenicity study alone or adsorbed to aluminum hydroxide. Differently from GMMA which display a modified LPS, *Nm* OMV were obtained by extraction with deoxycholate detergent and therefore wild type LPS was largely removed. When OMV were adsorbed to aluminum hydroxide, the pyrogenicity was reduced four-fold in rabbits and this reduction was also observed in the Limulus Amebocyte Lysate (LAL) test. However, when reactogenicity was evaluated in human volunteers, the adsorbed vaccine gave more local side effects and longer duration of tenderness at the site of injection than the unadsorbed vaccine, whereas no differences were observed between the two formulations in terms of minimal, unspecific systemic reactions (53). Therefore, more studies in humans are needed to better elucidate the contribution of Alhydrogel to reactogenicity.

Preclinical studies in mice showed that immunization with *S*. Typhimurium GMMA not adjuvanted with Alhydrogel elicited antigen-specific IgG titers higher than after immunization with GMMA with adjuvant. For *S*. Enteritidis and *S. flexneri* 6 GMMA, addition of Alhydrogel did not increase or decreased anti-O-antigen-specific IgG response (13, 40). On the opposite, a preclinical study with *Nm* OMV showed that the addition of aluminum hydroxide improved antigen-specific IgG levels and bactericidal activity (54). Differences in the LPS content and structure in GMMA and detergent extracted OMV can have an impact on innate immunity activation and therefore on self-adjuvanticity. More studies will be needed to evaluate the added value of Alhydrogel or alternative adjuvants on GMMA immunogenicity.

GMMA vaccines are highly immunogenic, but there are no many insights into their mechanism of action. Few experiments have been performed so far to understand what happens at the injection site and after injection, and to identify the factors which influence the cellular and humoral responses elicited.

It has been shown that after injection, nanoparticles are coated by proteins present in the interstitial fluid (55). The nature and amount of these proteins is influenced by their particle size and physico-chemical properties that may result in augmented or reduced interaction with immune cells. No information is available so far for GMMA, and it may be important to consider that GMMA from different bacteria can display LPS with different charge, which can in turn influence the quality of the interstitial fluid proteins binding on their surface. Indeed, net charge and hydrophobicity are some of the several physical–chemical features of nanoparticles which influence their interactions with plasma proteins (56).

**Figure 1:**
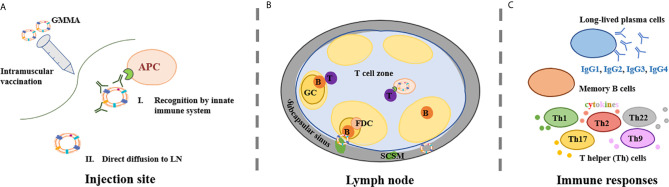
GMMA mechanism of action. **(A)** GMMA vaccines have been administered intramuscularly. At the injection site, GMMA may be coated by proteins present in the interstitial fluid. The kind and amount of these proteins is influenced by GMMA size and physico-chemical properties. GMMA trafficking to the lymph node (LN) may require cellular transport from antigen presenting cells (APCs) **(A-I)** but GMMA can also be able to drain freely to the LN **(A-II)**. **(B)** Upon arrival into the LN, GMMA and APCs circulate into the LN subcapsular sinus where subcapsular sinus macrophages (SCSM) reside. SCSM can uptake GMMA in complex with IgG and can reach the LN follicular area where they transfer the antigen to B cells. B cell receptor crosslinking and B cell activation is facilitated by repetitive display of optimally spaced antigen on GMMA. Also follicular dendritic cells (FDCs) play a major role in germinal center (GC) kinetics and in the development of humoral responses. GMMA delivered to the LN *via* active transport are presented to naïve T cells with antigen-cognate T cell receptors in the T cell zone. T cells also interact with activated B cells, that present them antigen-derived peptides thus providing T cell ‘help’ to B cells, sustaining their activation and driving the formation of GCs. **(C)** GMMA drive the selection of B cell clones with high antigen affinity into long-lasting memory B cells and/or long-lived antibody-producing plasma cells and possibly the differentiation of T cells in different subsets of T helper (Th) cells.

At the injection site, GMMA encounter tissue resident immune cells and dendritic cells (DCs) recruited at the site through the inflammation process (**Figure 1A-I**). GMMA uptake by DCs should favor DC maturation, allowing trafficking to the lymph node (LN), but this interaction has not been proven so far. It is known that OMV are able to drive DC maturation. Indeed, native OMV derived from the facultative-intracellular bacterium *Burkholderia pseudomallei* were shown to favor DC maturation and activation both *in vitro* and *in vivo* in a mouse model (57). Moreover, Schetters et al. demonstrated that the LPS present on OMV was able to induce, through the myeloid differentiation primary response 88 (Myd88) adapter protein, maturation of human monocyte-derived DCs, murine bone marrow-derived DCs and CD11c+ splenic DCs (58).

Since GMMA are 25-250 nm vesicles, their size should allow them direct diffusion into lymphatic vessels (**Figure 1A-II**). It has been shown that nanoparticles of 20–200 nm and 30 nm virus-like particles (VLPs) are detected in LN resident CD8+ DCs and macrophages two hours after vaccination, thus indicating that they can be rapidly drained to the LN without active transport by antigen presenting cells (APCs) (59). As particle size increases over 200 nm, antigens are likely to require APCs for trafficking to the LNs.

Upon arrival into the LN, nanoparticulated antigens like GMMA and cells circulate into the LN subcapsular sinus where subcapsular sinus macrophages (SCSM) reside (**Figure 1B**). SCSM can uptake GMMA in complex with IgG and can reach the LN follicular area where they transfer the antigen to B cells *via* CR2 (60). The GMMA-IgG complexes allow B cell receptor crosslinking with the CR2 complex thus lowering the affinity threshold for B cell activation (61). B cell receptor crosslinking is facilitated by repetitive display of optimally spaced antigen on GMMAs. The antigen is also transferred to follicular dendritic cells (FDCs), which play a major role in the development of humoral responses. Indeed, FDCs secrete the B cell attracting chemokine CXCL13 which induces activated B cell migration into the GC, produce B-cell activating factor (BAFF), which regulates GC B cell survival and retain the antigen for long time, thus enabling persistent GC reactions and resulting in long-lasting plasma cells that secrete high affinity antibodies (62). VLPs rapid delivery to FDCs was proven to be dependent on natural IgM and complement (60), and their ability to facilitate antigen persistence in the LN shown in comparison to soluble antigens (63). Besides many similarities between VLPs and GMMA, no data are available so far for GMMA. If GMMA are delivered to the LN *via* active DC transport, the outer membrane antigens are presented to naïve T cells with antigen-cognate T cell receptors in the T cell zone. Moreover, T cells also interact with activated B cells, presenting them antigen-derived peptides thus providing T cell ‘help’ to B cells, sustaining their activation and driving the formation of GCs (64).

Preclinical and clinical studies conducted with GMMA-based vaccines have addressed humoral responses but poorly the cellular response (**Figure 1C**). Baker and co-authors demonstrated that OMV could induce robust cellular immune responses that exceeded those induced by a live-attenuated strain (65).

GMMA could also be used as adjuvants and as alternative carrier. When OMV have been tested as adjuvants for heterologous peptides or ovalbumin, they elicited broad antigen-specific immune responses, including antibody, B cells, CD4 T cells, and CD8 T cells. Humoral immune responses to ovalbumin elicited upon immunization with OMV were higher compared to those induced by ovalbumin adjuvanted with Alhydrogel and CpG DNA (57). When used as carrier, GMMA have shown superior immunogenicity compared with conventional carrier proteins such as CRM_197_ (13, 40, 44). It will be interesting to understand the immunological mechanisms behind this superiority.

## Future Directions

GMMA is recognized as a powerful vaccine technology. It has the unique advantage to be a multivalent vaccine with a straightforward production process and simple purification steps to generate high yield, and which does not need the addition of detergents as in the case of classical OMV purification which may alter vaccine composition and antigen conformation. The presentation of multiple antigens in their natural environment and the inherent GMMA characteristics are the driving force of the high immunogenic profile.

However, there are still many immunogenicity aspects that need to be unraveled for a deep understanding of the full potential of GMMA-based vaccines. We have recently started to investigate more in depth the impact that GMMA structural features may have on the immune response. One of these features is the saccharide length, a well-known parameter that can modulate the intensity and quality of immune response elicited by glycoconjugate vaccines. In contrast with what observed for traditional glycoconjugates, O-antigen length did not result to be a critical parameter for GMMA immunogenicity, independently by the pathogen and by the sugar structural characteristics (11). Moreover, we demonstrated that exposure of GMMA to high-temperature (100°C for 40 min) did not impact GMMA stability and immunogenicity in mice, whereas milder temperatures for a longer period of time did (37°C or 50°C for 4 weeks). Major differences were related to O-antigen O-acetylation and its release from vesicles (66). Although what makes GMMA a unique antigen and delivery system is known, the mechanism of action *in vivo* is still unknown. Therefore, more efforts should be directed towards the dissection of what happens at the GMMA injection site and afterwards and the mechanisms which mediate the immune response, either in presence or absence of an adjuvant. Unravelling such mechanisms will allow to improve the design of GMMA-based vaccines. Clinical studies conducted so far with GMMA-based vaccines have confirmed the good immunogenicity and safety profile observed in preclinical studies. However, even though GMMA technology has been applied to several bacterial targets in preclinical studies, only few of them have progressed into clinical trials in adults but none in children and infants. More clinical data will be needed to corroborate the preclinical findings, and further evaluation of GMMA-based *Shigella* and *Salmonella* vaccines are expected to provide a better understanding of the immunological potential of this platform.

## Author Contributions

All authors contributed to the article and approved the submitted version.

## Funding

This work was undertaken at the request of and sponsored by GlaxoSmithKline Biologicals SA.

## Conflict of Interest

This work was undertaken at the request of and sponsored by GlaxoSmithKline Biologicals SA. GSK Vaccines Institute for Global Health Srl is an affiliate of GlaxoSmithKline Biologicals SA. All authors are employed by the GSK group of companies.

## Publisher’s Note

All claims expressed in this article are solely those of the authors and do not necessarily represent those of their affiliated organizations, or those of the publisher, the editors and the reviewers. Any product that may be evaluated in this article, or claim that may be made by its manufacturer, is not guaranteed or endorsed by the publisher.
